# Combining Blink, Pupil, and Response Time Measures in a Concealed Knowledge Test

**DOI:** 10.3389/fpsyg.2012.00614

**Published:** 2013-02-04

**Authors:** Travis L. Seymour, Christopher A. Baker, Joshua T. Gaunt

**Affiliations:** ^1^Cognitive Modeling Laboratory, Psychology Department, University of California Santa CruzSanta Cruz, CA, USA

**Keywords:** deception, guilty knowledge, concealed information, lying, pupil, blinks, recognition

## Abstract

The response time (RT) based *Concealed Knowledge Test* (CKT) has been shown to accurately detect participants’ knowledge of mock-crime-related information. Tests based on ocular measures such as pupil-size and blink-rate have sometimes resulted in poor classification, or lacked detailed classification analyses. The present study examines the fitness of multiple pupil and blink related responses in the CKT paradigm. To maximize classification efficiency, participants’ concealed knowledge was assessed using both individual test measures and combinations of test measures. Results show that individual pupil-size, pupil-slope, and pre-response blink-rate measures produce efficient classifications. Combining pupil and blink measures yielded more accuracy classifications than individual ocular measures. Although RT-based tests proved efficient, combining RT with ocular measures had little incremental benefit. It is argued that covertly assessing ocular measures during RT-based tests may guard against effective countermeasure use in applied settings. A compound classification procedure was used to categorize individual participants and yielded high hit rates and low false-alarm rates without the need for adjustments between test paradigms and subject populations. We conclude that with appropriate test paradigms and classification analyses, ocular measures may prove as effective as other indices, though additional research is needed.

## Introduction

### Combining blink, pupil, and response time measures in a concealed knowledge test

Researchers have developed several paradigms to assess whether or not participants are concealing sensitive information (for reviews, see Ben-Shakhar and Furedy, 1990; Lykken, 1998; MacLaren, 2001; Ben-Shakhar and Elaad, 2003). This approach differs from the control questions “lie detector” test because it focuses on the ability of various dependent measures to indicate when participants recognize critical information as opposed to lying about it *per se*. A meta-analysis of concealed knowledge tests (CKT) revealed an average hit rate of 0.83 and a false-alarm rate of 0.04 (Ben-Shakhar and Elaad, 2003). In light of the dubious theoretical underpinnings and highly variable performance of the traditional “lie detector” test (National Research Council, 2003), many researchers have developed tests using indices of concealed knowledge, rather than indices of deception (c.f. Verschuere et al., 2011).

### Concealed knowledge detection

Following previous work by Rosenfeld et al. (1988), Farwell and Donchin described a CKT paradigm in which responses to familiar crime-related *probes* could be compared to familiar *target* items not associated with the crime (Farwell and Donchin, 1991). Participants memorized a set of probe phrases (e.g., “White Shirt”) and then used this information to enact a mock-crime scenario. Later, they memorized a set of target phrases (e.g., “Blue Coat”) unrelated to the scenario. In a subsequent memory test, participants accurately indicated their recognition of target phrases, but denied recognition of familiar-probe phrases. On trials containing novel *irrelevant* phrases, participants accurately indicated their lack of knowledge. The target stimuli in this paradigm are important because only they require an affirmative response. Without targets, one could respond “no” on each trial without considering the stimulus; a strategy that could attenuate the effectiveness of the test (for an alternate view, see Rosenfeld et al., 2006). Thus, targets force participants to process each stimulus (including crime-relevant probes). Using evoked-related brain potentials (ERP) to index stimulus familiarity in the brain’s anterior cingulate cortex, Farwell and Donchin achieved a hit rate of 0.9 with no false-alarms.

Using a similar paradigm (but with a 1000 ms response deadline), Seymour et al. (2000) examined whether response time (RT) and accuracy were sufficient to detect concealed knowledge from a mock-crime. Results showed that “no” responses to crime-related probes were significantly slower and less accurate than to unfamiliar irrelevant items. A specialized individual classification procedure that compared participants’ probe and irrelevant RT distributions led to a 0.93 hit rate with no false-alarms. Similar results have been reported in subsequent studies using related CKT test procedures and analyses (Seymour and Kerlin, 2008; Seymour and Fraynt, 2009; Verschuere et al., 2010; Visu-Petra et al., 2011).

Although the RT-based CKT can yield high detection rates, examinees may attempt to manipulate their responses to undermine a test’s effectiveness. Studies have shown that a variety of physiological and neuropsychological-based tests are susceptible to strategic countermeasures that reduce detection rates (Seymour and Kerlin, 2008; Seymour and Fraynt, 2009; Verschuere et al., 2010; Visu-Petra et al., 2011). For the RT measure in the CKT paradigm, results have been mixed. Some data suggest that attempting to appear unfamiliar with familiar-probes by equating probe and irrelevant RTs is generally ineffective (Seymour et al., 2000). However, effective countermeasures have been demonstrated using CKT paradigms without response deadlines (e.g., Rosenfeld et al., 2004), and emotional Stroop (Williams et al., 1996) based detection paradigms (Gronau et al., 2005; Degner, 2009).

One approach that may potentially lead to more accurate countermeasure-resistant paradigms involves simultaneously assessing multiple measures in a single paradigm (Gronau et al., 2005). Although previous work has examined in detail the anti-countermeasure benefits of combining various polygraph-based measures (respiratory rate, heart-rate, electrodermal response, etc.; c.f., Elaad, 2011), few have included RT and ocular measures. However, some studies have examined such measures in combined tests. Cutrow et al. (1972) reported that an amalgamation of respiratory rate, eye blink-rate, pulse, and electrodermal responses allowed differentiation between answers to mock-crime and irrelevant questions. However, classification analyses were omitted. Without individual classification rates (in particular false-alarms), this result cannot be properly evaluated. Allen et al. (1992) also analyzed a CKT using combined measures (ERP and RT) that yielded average hit rates of 0.98 and false-alarm rates of 0.03. Although the combined-measure false-alarm rate was only 0.02 greater than using ERP alone, the addition of RT reduced the miss rate by 0.04. Several studies have examined combinations of polygraph measures such as electrodermal response, heart-rate, and respiratory rate. Such combined tests often yield small but robust improvements over individual indicators (e.g., Elaad et al., 1992; Gamer et al., 2008). However, in other studies, such combinations have failed to outperform their individual counterparts (e.g., Bradley and Warfield, 1984; Verschuere et al., 2007). Although differences between studies may explain this disparity (c.f. Meijer et al., 2007), in the present study we examined the benefit of combining ocular and RT-based measures of concealed knowledge.

### Ocular measures of concealed knowledge

A potentially effective test may combine intentional motor responses such as RT with more autonomic ocular responses such as pupil-size and blinking rate; both of which can be assessed simultaneously without interference. Modern eye-trackers can be calibrated and used without participants’ awareness, limiting opportunities for countermeasures. Even with conspicuous eye measurement, automatic responses such as blinking and pupil dilation may be difficult to control systematically in a covert fashion. Of course, the advantage of combining ocular and RT measures depends on the degree to which these measures are correlated with one another. One reason why consistently successful combined paradigms have been elusive is that the diagnostic accuracy of individual ocular measures remains uncertain (c.f. Gamer, 2011). For example, one potential ocular measure, internally cued (i.e., endogenous) blinking, is typically correlated with cognitive demand (unlike reflexive or voluntary blinks) (Drew, 1951; Holland and Tarlow, 1972; Bagley and Manelis, 1979; Stern et al., 1984; Bauer et al., 1987; Goldstein et al., 1992). Accordingly, they tend to be inhibited during the processing or anticipation of relevant stimuli and occur most frequently at junctures between processing. Peak blink-rate (maximum average blink-rate reached during each trial) tends to increase as a function of processing load whereas latency to peak rate (average time required on each trial to reach that trial’s peak blink-rate) increases with processing duration (Stern et al., 1984; Bauer et al., 1987; Goldstein et al., 1992; Ichikawa and Ohira, 2004). Some studies have shown that overall blinking behavior is sensitive to concealed knowledge (Janisse and Bradley, 1980; Dionisio et al., 2001; Fukuda, 2001; Leal and Vrij, 2008). For example, Leal and Vrij (2010) examined blink activity during a paradigm in which participants made either truthful or deceptive statements about participation in a mock-crime. Results showed that liars displayed significantly fewer blinks for probe questions than for controls. Truth tellers showed no such difference. A discriminant analysis on probe-control differences for each participant yielded a 0.75 hit rate and a 0.23 false-alarm rate.

In addition to overall blink-rate, it has been suggested that temporal variations in blink activity can differentiate probe and irrelevant stimuli and perform significantly better than overall blink-rate (Stern et al., 1984; Fukuda, 2001; Ichikawa and Ohira, 2004; Leal and Vrij, 2008). For example, Fukuda (2001) measured the number of blinks participants produce on each trial during a concealed knowledge paradigm and plotted them as a function of trial duration. Analysis was done on the shape of the resulting temporal distribution of blinking (TDB) and assessed various characteristics such as average blink-rate, peak blink-rate, and time-to-peak. Results showed that responding to probe stimuli led to a higher average blink-rate that peaked earlier and higher than to irrelevant stimuli. Unfortunately, a detailed classification analysis was omitted making it difficult to assess the diagnosticity of the TDB measure. Nevertheless, a successful blinking measure might prove an important addition to a combined-measure CKT. Crucially, Goldstein et al. (1992) found that RT and blinking were uncorrelated and influenced by different task variables, suggesting that these measures may be ideal candidates for combined tests.

Similar to blinks, pupil-size has been shown to reliably index cognitive task demand (Beatty, 1982; Steinhauer and Hakerem, 1992; Karatekin et al., 2004), and has also been shown to index emotional arousal (Bradley et al., 2008). Because of such results, pupil-size has been explored as a measure of deception (Berrien and Huntington, 1942; Heilveil, 1976; Janisse and Bradley, 1980; Lubow and Fein, 1996; Dionisio et al., 2001; Webb et al., 2009a,b). Fluctuations in pupil-size can be highly reliable even when small in magnitude, with researchers reporting robust effects as small as 0.1 mm (Hakerem and Sutton, 1966) and 0.015 mm (Beatty, 1988). Lubow and Fein (1996) found greater pupil dilation following presentation of mock-crime-related probes than irrelevant items in a CKT paradigm. A classification analysis yielded hit rates of 0.50 and 0.70 with no false-alarms (overall detection accuracies of 75 and 85%). This was an improvement on an earlier pupil-based test reporting overall detection accuracies between 66 and 69% (Janisse and Bradley, 1980). A later study by Dionisio et al. (2001), in which participants made true and then false statements about benign scenarios, reported greater average pupil-size during false than true statements for 92% of participants. Again, the necessary classification information (false-alarm rates in particular) was unavailable for this study, as well as the Janisse and Bradley studies. Cook et al. (2012) did report detailed classification results from a test consisting of true/false questions, e.g., “I took the $20 from the secretary’s purse.” Both pupil-size and eye scan-patterns were recorded. Across two experiments, they found an average hit rate of 0.80, and an average false-alarm rate of 0.13. Kircher et al. (2010) reported results from tests using demographic and true/false questions. Deception was indexed using pupil-size, reading pattern, and RT measures, but average hit rate (0.80) and false-alarm rate (0.15) were similar to Cook and colleagues. Overall, pupil-based measures seem promising for the CKT paradigm, but more work is needed to find robust methods that increase hit rates and reduce false-alarm rates to levels comparable with other more established CKT measures.

### A new test combining behavioral and ocular measures

The lack of detailed individual classification analyses limits the ability to assess ocular measures in some CKT studies. In the present study, this is remedied by examining both pupil-size and blink measures using an individual subject classification procedure for participants familiar with probes (to assess hit and miss rates) and participants unfamiliar with probes (to assess correct-rejection and false-alarm rates). Another question inconsistently answered in the literature is the fitness of combined-measure CKT paradigms. Although work exists showing successful combinations of polygraph measures (e.g., Gamer et al., 2008), consistent results are not available for combinations of ocular and RT measures. Although this disparity may be in part due to differences in test parameters or classification analyses, we argue that combinations of more disparate measures could be more diagnostic, and could potentially thwart the use of some countermeasures. To our knowledge, this is the first study to evaluate the combined diagnosticity of response-time, pupil-size, and blink measures. In addition to the standard mean pupil-size and peak blink-rate measures, we added Fukuda’s (2001) blink distribution measures and a new pupil-slope measure following observations by Lubow and Fein (1996). To require that participants process each stimulus, we used the 3-stimulus variant of the CKT (probe = “no,” target = “yes,” and irrelevant = “no”).

## Materials and Methods

### Participants

Sixty undergraduate students (67% female) at the University of California Santa Cruz participated in the experiment for course credit. All participants had normal or corrected-to-normal vision.

### Materials and apparatus

The stimuli were 66 luminance-matched color pictures of non-familiar human faces (half female) with neutral expressions taken from the Aberdeen Psychological Image Collection (Hancock, 2004). Pictures were presented on a 17″ monitor with a refresh rate of 85 Hz and each subtended an area of 12.5 × 16.2° of visual angle at a viewing distance of 18″. Stimulus presentation and randomization, as well as the recording of RT and accuracy were managed using E-Prime presentation software (Schneider et al., 2002). RTs were entered on a Cedrus four-button response pad (Cedrus Corporation, San Pedro, CA, USA). An Arrington ViewPoint eye-tracker (Arrington Research, Inc. Scottsdale, AZ, USA) was used to record blinking and pupil-size at a sample rate of 60 Hz. Participants’ heads were stabilized using a chin rest. During calibration, the location and extent of participants’ right pupil and the location of their pupil glint were mapped. The best fitting ellipse was constantly computed to fit the pupil over time. Pupil-size is thus an online measure in millimeters of the transverse diameter of this ellipse. Blinks were also measured with respect to this geometry. When participants blink, their eyelid falls and the best fitting ellipse becomes increasingly flat before the pupil disappears altogether. This transition is used to detect blinks, but requires a threshold value. Pupil geometry is partially a function of viewing angle with respect to the display and the position of the eyes; thus, the exact height to width ratio of the ellipse that will indicate a blink must be determined separately for each participant. To achieve this, the range of aspect ratios noted during spatial calibration (participants cued to look at various points across the display) was recorded. Subsequently, a blink threshold was chosen for each participant to distinguish between real blinks and flattened ellipses that occurred naturally when eyes were moved toward the various edges of the display. The mean threshold ratio was 0.6.

### Design and procedure

The experiment was comprised of a series of tasks to be completed in the following order: A probe-learning phase, a retention interval, a target-learning phase, and a picture recognition task. Each session lasted approximately 1 h.

#### Probe-learning phase

For each participant, a set of six probe faces was selected randomly from the entire pool of faces. The study procedure for probe faces was designed to ensure elaborative encoding of probe stimuli (c.f., Seymour and Kerlin, 2008). This is in contrast to mock-crime procedures during which individual variations in memory, motivation, and attention can lead to the encoding of some probe items but not others (Carmel et al., 2003). Such variations may increase potential external validity, but could lead to the confounding of mock-crime effectiveness and the diagnostic accuracy of the test (Seymour and Fraynt, 2009).

Participants studied each face for 45 s and were then shown one of six facial-feature questions (e.g., “did that person have facial hair?”). These questions were chosen randomly with replacement to prevent anticipation. After each feature judgment, the face was shown again for a mirror image judgment. Each image was either flipped on its vertical axis or not flipped at all. Participants pressed one button for “same” and another for “mirror” and were given immediate accuracy feedback. This cycle, in which face image study is followed by feature and mirror judgments, was repeated for each of the six probe faces. Once this cycle had been completed for all six probes, the order of faces was re-randomized and the study process was repeated until the entire set of probes was studied a total of three times. After this portion of the probe-learning phase was completed, participants were asked to rate each picture for its perceived attractiveness (seven-point Likert scale), honesty (seven-point Likert scale), and age (open ended).

#### Retention interval

To prevent rehearsal of probe items during the 10 min retention interval, participants completed a set of difficult mathematical word problems (taken from Patalano and Seifert, 1994).

#### Target-learning phase

Following the retention interval, six additional faces were randomly selected to be target stimuli. Targets were studied in the same manner as probes. That is, faces were shown individually for study and followed by both feature and mirror judgments. However, for targets there were no attractiveness, honesty, or age ratings. This study difference affords participants a basis on which to distinguish probe and target faces in the subsequent recognition task (Seymour and Kerlin, 2008).

#### Picture recognition task

Before beginning the recognition task, participants’ gaze coordinates were mapped to a standardized space via an eye-tracking calibration procedure. Following calibration, participants were shown a series of pictures and made speeded recognition judgments. On each trial, participants first saw a white visual mask with a black fixation-cross displayed at its center. After 1200 ms, a stimulus picture replaced the mask and remained on the screen until a response was made. Participants were asked to indicate on each trial their familiarity with the stimulus. For target faces they were to truthfully press a button marked “yes.” Similarly, for irrelevant faces, participants were to truthfully respond “no.” However, for probe faces participants were asked to deceptively respond “no,” despite their actual familiarity with these stimuli. Note that although participants were told that they were completing a deception task and that success meant responding just as quickly and accurately to probe stimuli as they did to irrelevant stimuli, no specific countermeasure instructions or monetary incentives were offered. After each response a blank screen was shown before the next trial began for a random duration between 2000 and 2500 ms. A 3000 ms deadline was used; responses longer than the deadline were followed by an “ERROR: TOO SLOW” warning. Otherwise, no feedback was given during each block. In previous studies using this paradigm with two-word verbal phrases, deadlines of 1000 ms (Seymour et al., 2000) and 1500 ms were used (Seymour and Kerlin, 2008; Seymour and Fraynt, 2009). The use here of a 3000 ms deadline was necessary given the relative complexity and high feature overlap of face stimuli (Bruce, 1982).

Each trial block contained one presentation of each face picture in the stimulus set (six targets, six probes, and 24 irrelevants) in a new random-order, for a total of 36 trials. Participants were randomly assigned to either a *familiar-probe* condition (in which probes were previously studied faces) or an *unfamiliar-probe* condition (in which probes were new faces). To participants, unfamiliar-probes are essentially irrelevants; this condition is analogous to testing an unaware examinee and is used to estimate the test’s false-alarm rate. Following each block, participants were shown a feedback screen including mean accuracy and the number of “Too Slow” errors for that block. In each condition, three blocks were completed for a total of 108 trials per participant.

### Analyses and predictions

#### Individual and combined test measures

Prior to each individual measure’s analysis, we calculated within-subject *Z*-scores to give a better indication of the effect size for each measure uncontaminated by individual differences in general responsiveness (c.f. Ben-Shakhar, 1985). In particular, for each participant we calculated the mean of all that participant’s responses (regardless of stimulus type), and subtracted this value from each score prior to dividing this result by the SD of all of that participant’s responses (regardless of stimulus type). Although all analyses and figures represent standardized data, Table 1 lists the mean untransformed data for each measure. Although Table 1 lists the mean and SD for each stimulus type, only probe and irrelevant stimuli were used for statistical analyses and classification. For the classification of individual participants’ data, both individual and combined measures were used. Combined measures were simple sums of individual measures.

**Table 1:** **Mean un-standardized data by stimulus type and condition for each measure**.

Measure	Stimulus type			Effect
	Irrelevant	Probe	Target	
**FAMILIAR-PROBE CONDITION**				
Response time (ms)	740 (150)	1086 (269)	908 (142)	346
Accuracy (%)	98 (3)	69 (24)	84 (15)	29
Pupil-size (mm)	3.9 (0.51)	4.0 (0.52)	4.0 (0.51)	0.10
Pupil-slope (×1000 mm)	0.49 (0.11)	0.59 (0.14)	0.53 (0.13)	0.10
Peak blink-rate (b/s)	0.09 (0.23)	0.27 (0.49)	0.10 (0.19)	0.18
**UNFAMILIAR-PROBE CONDITION**				
Response time (ms)	792 (132)	757 (164)	828 (189)	−35
Accuracy (%)	97 (4)	98 (3)	87 (13)	−1.0
Pupil-size (×1000 mm)	4.1 (0.49)	4.1 (0.50)	4.1 (0.48)	0
Pupil-slope (mm)	0.46 (0.15)	0.46 (0.18)	0.48 (0.18)	0
Peak blink-rate (b/s)	0.30 (0.95)	0.37 (1.3)	0.40 (1.7)	0.07

*SDs are indicated with parenthesis. Effect calculations involve subtracting irrelevant from probe responses, except for accuracy, which is irrelevant – probe*.

The Eta-squared statistic is included for each analysis as a measure of effect size. All *post hoc* *t*-tests were compared against a Tukey HSD corrected alpha level, and all *t*-tests were treated as *post hoc* unless otherwise noted. Lastly, all statistical tests were compared against a nominal alpha level of 0.05 unless otherwise noted.

#### Response time and accuracy

For RT and accuracy measures, we compared probe and irrelevant distributions as a function of the two probe-familiarity conditions. For the RT measure, only correct trials were included in the analysis. As in previous research using the present paradigm, we expected that Probes would be slower and less accurate in the familiar-probe condition compared to the unfamiliar-probe condition (e.g., Allen et al., 1992; Seymour and Kerlin, 2008; Seymour and Fraynt, 2009; Verschuere et al., 2010; Visu-Petra et al., 2011).

#### Blinking measures

Following Fukuda (2001), we analyzed endogenous blink-rate as a function of probe-familiarity condition and stimulus type for correct trials. The analysis window was divided into 25, 50 ms bins and a TDB was computed for each participant. Blink-rate was calculated for each bin by dividing the total number of blinks for that bin and stimulus type by the total number of trials for that stimulus type. The resulting value (i.e., blinks per 50 ms) was then multiplied by 20 for conversion into blinks-per-second (c.f. Fukuda, 2001) prior to being converted to *Z*-Scores. The resulting TDBs, averaged over participant, are plotted in Figure 2 by condition. Fukuda reported significant inhibition throughout most of the time the stimulus was onscreen. However, in the period just prior to the response, a significant increase in blinking occurred on probe trials only. Thus, we predicted that pre-response blink-rate would be likewise diagnostic in the current study. To identify the appropriate region for analysis, we examined blinking behavior across each trial over all stimulus types. Similar to Fukuda, participants in the current study rarely blinked during stimulus presentation. Out of the 5616 available correct trials, only 151 (2.6%) contained blinking during the first 400 ms following stimulus onset. In contrast, during the period from 400 ms prior to stimulus offset (i.e., response initiation) to 800 ms after stimulus offset we recorded 3108 trials with blinking (55%). This is typical for blinking behavior, which tends to occur between processing stages rather than during those stages. Thus, blinks were analyzed for this 1250 ms window relative to stimulus offset.

In addition to greater mean blink-rate for probes just prior to the response, and an even larger one afterward, Fukuda (2001) also found similar differences between familiar-probe and irrelevant items using peak blink-rate and time-to-peak blink-rate measures. Thus, we predicted that each of these four sub-measures of the TDB would also show greater blinks-per-second for probes than irrelevants in the familiar-probe condition only. If the TDB during the familiar-probe condition contains the numerous deviations predicted here, then we would also expect that the entire TDB function (binned blinks over time) for probes would differ significantly from the irrelevant TDB during the probe condition only. Thus, we also analyzed TDB as a function of condition. If diagnostic, classification on this function alone may be preferable to classification based on various individual components.

#### Pupil measures

**Figure 1:**
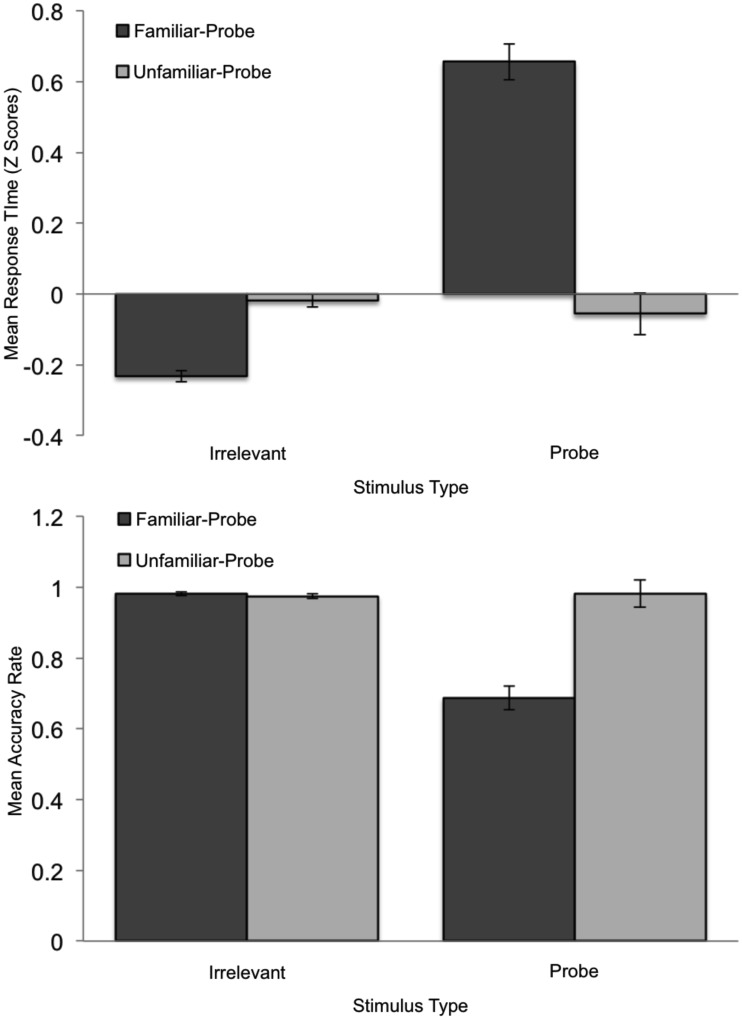
**Mean standardized RT (top graph) and Accuracy (bottom graph) plotted as a function of Stimulus Type and Condition**. Error bars indicate standard error of the mean.

Based on prior research described earlier, we predicted that mean pupil-size would be greater on probe than irrelevant trials in the familiar-probe condition. However, Lubow and Fein (1996) also observed increased pupil-slopes for familiar-probe stimuli. Although slope was not analyzed, this effect was visually apparent in their graphs. Thus, we predicted that pupil-size would not only be greater on average for familiar-probes than irrelevants, but would grow faster over time. Pupil-slope was computed by fitting a least-squares regression line through each trial’s pupil data (stimulus onset to response) and then computing the change in pupil-size over time represented by this line. Both mean pupil-size and pupil-slope measures were computed over pupil data from the first 1500 ms of each trial following stimulus onset. Figure 4 depicts the mean standardized pupil data as a function of stimulus type and time during this period. Because in the current paradigm stimulus offset is concomitant with the response, this visual representation is sub-optimal; although probes and fillers are represented throughout this range, toward the end there is a greater proportion of probe than irrelevant responses (c.f., mean RT pattern in Figure 1; Table 1).

### Classification rationale and procedure

Overall mean differences between probe and irrelevant responses are not sufficient conditions for successful diagnostic tests. Often, CKT classification procedures consider the range of test outcomes (e.g., differences between probe and irrelevant responses), choose a cutoff value that maximizes the differentiation between these responses in the studied sample (e.g., the median value), and then report the resulting classification results using this cutoff (e.g., Farwell and Donchin, 1991; Lubow and Fein, 1996). A popular alternative method is to derive the optimal cutoff based from a receiver operating characteristic (ROC) analyses (Green and Swets, 1966; Bamber, 1975; Hanley, 1982), which includes an analysis of the tradeoff between a test’s hit and false-alarm rates over a series of cutoffs. A poor test (efficiency near 0.5) is one in which hits and false-alarms are perfectly related so that a cutoff change that achieves a 1% increase in the hit rate results in the same increase in the false-alarm rate. An efficient test (efficiency near 1) allows the maximization of hit rate with minimum increases in false-alarm rate. Thus, ROC analysis offers a better understanding of the fitness of the test under investigation across a variety of cutoffs. To classify a group of responses from a CKT procedure, the cutoff that maximizes hit rate and minimizes false-alarm rate can be chosen and applied to the data.

Other classification approaches for CKT data that may involve determining cutoff points include maximum rank analysis (e.g., Lykken, 1959; Bradley and Warfield, 1984), discriminant-function analysis (Nose et al., 2009), and logistic regression analysis (Gamer et al., 2006; Gamer, 2011). The primary advantage of such techniques is their ability to model the relationship between the predictor variables and test outcomes (e.g., guilty vs. innocent). The resulting discriminant-function is then used to calculate hit and false-alarm rates for the sample. This allows researchers to understand the discriminability of the sample under investigation, but may not give as clear a view of how well the discriminant-function will classify data from future tests. This is not a flaw in these methods, but requires that researchers either generate the classification model on a subset of available data and use it to predict the remaining data, or use the entire dataset and use the same function for classification in subsequent tests (e.g., Bradley et al., 1996). The latter is particularly difficult to do successfully if subject demographics or test parameters change from test to test (e.g., stimulus modality, response deadline, response stimulus interval, etc.). Regardless of whether one classifies using median cutoffs, ranks, or one of the various methods of producing discriminant-functions, functions developed using existing participant data may need to be updated for successful classification of future participants. This is especially probable if subsequent participants or test paradigms differ significantly from those used to develop the classification function.

In the present study, we avoid this particular concern by not basing classification on observed differences between probe and irrelevant responses in the current dataset and paradigm, but on theoretical ways in which any two distributions of responses may vary when produced by different psychological processes. In this way, the classification remains constant across changes to subjects, test parameters, or diagnostic measures.

Following Seymour et al. (2000) we used a *compound classification procedure* (CCP) in which each participant’s distribution of probe RTs was compared to their irrelevant RT distribution. Seymour and colleagues used three separate statistical tests that evaluated whether response distributions differed with respect to (a) the number of response errors (Fisher’s exact test), (b) their shape or skew (Kolmogorov–Smirnov test), (c) and their variation of scores (variance-ratio test). It was assumed that relative to a distribution of unfamiliar irrelevant responses, a distribution of familiar-probe responses would contain more errors, would be less positively skewed, or would have a greater variance. It was further assumed that differences might emerge on all three tests, or some subset. Thus, a statistical difference on either test would lead to the conclusion that participants were familiar with probes (if accurate, a hit is recorded, otherwise it is a false-alarm). No statistical difference on any test indicated that participants were unfamiliar with probes (if accurate, a correct-rejection, otherwise a miss). Using the three-test CCP, Seymour et al. achieved hit rates of 0.98 and 0.93, and false-alarm rates of 0.02 and 0 using test alphas of 0.05 and 0.01, respectively. This analysis technique has no free parameters and allows data produced by any continuous measure to be evaluated. The nominal alpha level required for each test’s significance is technically variable, however, it would be difficult to justify altering it beyond the standard 0.05 level. Due to the prohibitive nature of false-alarms in forensic contexts, it may be reasonable in some cases to reduce the level below 0.05 to make the test more conservative, but there is no more justification for increasing the alpha level above 0.05 than there would be for other statistical analyses in psychological research. Although Seymour and colleagues’ initial report used a verbal phrase based CKT, similar hit rates (0.91) and false-alarm rates (0.03) were achieved in a subsequent test using face pictures as stimuli (Seymour and Kerlin, 2008).

As in previous studies (Seymour et al., 2000; Seymour and Kerlin, 2008; Seymour and Fraynt, 2009), response accuracy in the present study successfully discriminates between probe and irrelevant responses in the familiar-probe condition. Despite this, we chose not to include accuracy in classification analyses because in previous studies where incentives were promised (Seymour et al., 2000; Seymour and Fraynt, 2009), the accuracy effect was significantly attenuated. Such attenuation has also been noticed in paradigms that offered no explicit incentive (e.g., Rosenfeld et al., 2004). Thus, although the diagnosticity of combined measures that include accuracy would likely be enhanced here, it is not believed that such benefits would extend to future studies using incentives, or applied contexts involving natural incentives. Thus each individual and combined measure was evaluated on the basis of distribution variance and shape, but the Fisher exact test for number of errors was not used.

In Seymour et al. (2000) each participant completed both familiar-probe and unfamiliar-probe tests thus serving as their own control for the classification analysis. In the present study, probe-familiarity was manipulated between subjects; data from participants in the familiar-probe condition were used to analyze hit and miss rates, and data from the unfamiliar-probe condition were used to assess false-alarms and correct-rejections. For each participant, probe and irrelevant response distributions were compared using each individual and combination of measures. Each comparison involved two statistical analyses; a variance-ratio test, and a Kolmogorov–Smirnov test. Thus, each participant’s probe and irrelevant response distributions were subject to 22 statistical comparisons (i.e., two statistical analyses for 11 individual and combined measures). For each participant’s statistical comparisons, a nominal alpha of 0.05 was assumed and Bonferroni corrected to 0.025.

Classification of each participant began with a variance-ratio test (also called the *F*-test for variances) to evaluate the one-tailed hypothesis that probe and irrelevant response distributions have different spreads. Subsequently, data were converted to overlapping cumulative distribution functions (normalized by sample size), and a Kolmogorov–Smirnov test (for review, see Kotz et al., 1983) was used to evaluate the one-tailed hypothesis that the cumulative probability at the maximum vertical deviation between the two curves, *D*, would be greater for probe than irrelevant distributions. The *D*-statistic ranges from 0 (no deviation) to 1 (maximal deviation). For sample sizes *n*_1_ (probe = 18) and *n*_2_ (irrelevant = 72), the corresponding *p*-value was determined by entering *D*/*S*(*n*) into a *D*-statistic table, where $s(n)=\sqrt{n_{1}+n_{2}/n_{1}n_{2}}.$ Values of 1.36 and 1.63 correspond to typical alpha levels of 0.05 and 0.01 and would require maximal deviations between distributions of 36 and 39% respectively. This statistic is particularly useful for comparing the shape of two response distributions because it is non-parametric. Also, unlike Student’s *t*-test, it does not make assumptions about the underlying distribution and is not influenced by changes in scale.

In the CCP, a “hit” results (probe knowledge indicated) if any 1 of the constituent tests’ null hypotheses is rejected. Lack of familiarity with probes is concluded only if neither test reaches statistical significance. A conservative threshold for significance balances the liberal nature of this rule. Bonferroni corrected alpha levels are used for each of the underlying statistical tests, so that a nominal alpha of 0.05 requires an actual difference between distributions at the *p* < 0.025 level. Additional care is warranted when comparing distributions that differ significantly in size, as is the case with each participant’s probe (*n*_1_ = 18) and irrelevant (*n*_2_ = 72) distributions. For example, if probe and irrelevant distributions each contained 15 very slow RTs, this might suggest that such RTs are not diagnostic and the fact that mean probe RT is greater than mean irrelevant RT is an artifact of the small probe sample. This spurious difference may also manifest itself in the variance-ratio and K–S statistics, leading to an increased false-alarm rate. To address this issue, a Fisher randomization procedure (Fisher, 1935) is used to verify any significant differences that result from K–S or variance-ratio tests. First a participant’s observed probe and irrelevant scores are pooled into one distribution of size *n*_1_ + *n*_2_. Then two new samples of sizes *n*_1_ and *n*_2_ are drawn without replacement and compared using the statistic of interest (K–S or variance-ratio, two tailed). After 1000 repetitions, if more than five statistical differences are found between these sampled distributions that equals or exceeds the original statistic for the observed distributions, the null hypothesis is accepted. The effect of this procedure is to essentially test how many probe-like responses are present in the observed irrelevant distribution. The more probe-like responses there are in the irrelevant distribution, the greater the chance of sampling a new probe distribution that is significantly different than a sampled irrelevant distribution using the statistic under investigation. If such a difference occurs more than 5 times out of 1000, the original statistical difference between the observed probe and irrelevant distributions is considered spurious and recorded as having been non-significant. Thus, although either of the constituent tests in the CCP may be used to determine probe-familiarity, the standard of proof is relatively high. One result of this conservatism is that the default classification is an unfamiliar-probe one.

The CCP is related to the parallel testing method (Appendix K, National Research Council, 2003) in that a set of predictors is assessed and a critical result on either test indicates the presence of some target condition (e.g., disease, guilty, etc.), and only non-significant results on all measures indicates the absence of the target condition. One difference is that in the parallel testing method, independent methods are ideally sought so that the inclusion of additional tests incrementally increases the hit rate of the method. Alternatively, the CCP was designed to assess various aspects of the same characteristic – the shape of the response distribution – achieved using variance-ratio and K–S tests. The goal of this overlap is to address complete or partial tradeoffs in participants’ responses to familiar-probe stimuli; they tend to be either more variable than irrelevants, more skewed than irrelevants, or both. A third test, Fisher’s exact, was previously used to address the final tradeoff observed whereby participants would trade speed for accuracy more in familiar-probe than irrelevant responses (c.f. Seymour et al., 2000). Although multiple correlated measures are not generally ideal when trying to minimize misses and false-alarms, the corrected alpha level required for each additional test in the CCP, and possibly the need to pass the Fisher randomization procedure, may counteract this concern. Indeed, it is possible that the combination of these constraints causes the test to be overly cautious. As a result, if the measure under investigation is not sufficiently diagnostic, both false-alarm rates and hit rates may be lowered. Ultimately, the true impact of the CCP on a test’s sensitivity and specificity would need to be modeled with statistical simulations. However, the low false-alarm rate and high hit rate previously reported using the CCP gives some indication that the low false-alarm rate does not come at the cost of an extreme number of misses.

## Results

Successful eye-tracking calibration of eight (13%) participants in the unfamiliar-probe condition was not possible. Thick eyeglasses, shifting contact lenses, and heavy applications of eyeliner make-up were among the most common obstacles. Thus, data from 52 participants (30 familiar-probe, 22 unfamiliar-probe) were included in the analysis.

### Omnibus tests

#### Response time and accuracy analysis

Response time data were submitted to a 2 Condition (familiar-probe vs. unfamiliar-probe) × 2 Stimulus Type (irrelevant, probe) mixed-model ANOVA with Stimulus Type as the within-subjects variable (see Figure 1, top graph). This analysis revealed main effects of Stimulus Type, *F*(1, 50) = 23.36, *p* < 0.001, η^2^ = 0.15, and Condition, *F*(1, 50) = 5.4, *p* = 0.02, η^2^ = 0.06, as well as a Condition × Stimulus Type interaction, *F*(1, 50) = 25.40, *p* < 0.001, η^2^ = 0.16. Participants in the familiar-probe condition took an average of 346 ms (SD = 218 ms) longer to respond “no” to familiar-probes than irrelevants, *t*(29) = 8.68, *p* < 0.001. In the unfamiliar-probe condition, participants could not distinguish probes from irrelevants and no differences emerged.

A similar analysis was performed on accuracy data and is also plotted in Figure 1 (bottom graph). This analysis revealed main effects of Stimulus Type, *F*(1, 50) = 20.62, *p* < 0.001, η^2^ = 0.16, and Condition, *F*(1, 50) = 8.51, *p* < 0.005, η^2^ = 0.08, as well as a Condition × Stimulus Type interaction, *F*(1, 50) = 52.73, *p* < 0.001, η^2^ = 0.33. Participants in the familiar-probe condition produced 29% (SD = 23%) more response errors to familiar-probe faces than irrelevant faces, *t*(29) = 6.92, *p* < 0.001. No such difference emerged in the unfamiliar-probe condition.

#### Blinking analysis

To assess the overall TDB by condition, A 2 Condition (familiar-probe vs. unfamiliar-probe) × 2 Stimulus Type (probe vs. irrelevant) × 25 Time (50 ms bins) mixed-model ANOVA was performed on TDB data with Stimulus Type and Time as within-subjects variables. There was a significant main effect of Time due to the increase in blinking 200–400 ms after the manual response, *F*(12.79, 639.60) = 28, *p* < 0.001, η^2^ = 0.29. Mauchly’s test indicated that the assumption of sphericity had been violated for this effect (ε = 0.35). Thus, degrees of freedom were corrected using Greenhouse–Geisser estimates. No other main effects or interactions were observed despite the large number of degrees of freedom available for this analysis.

**Figure 2:**
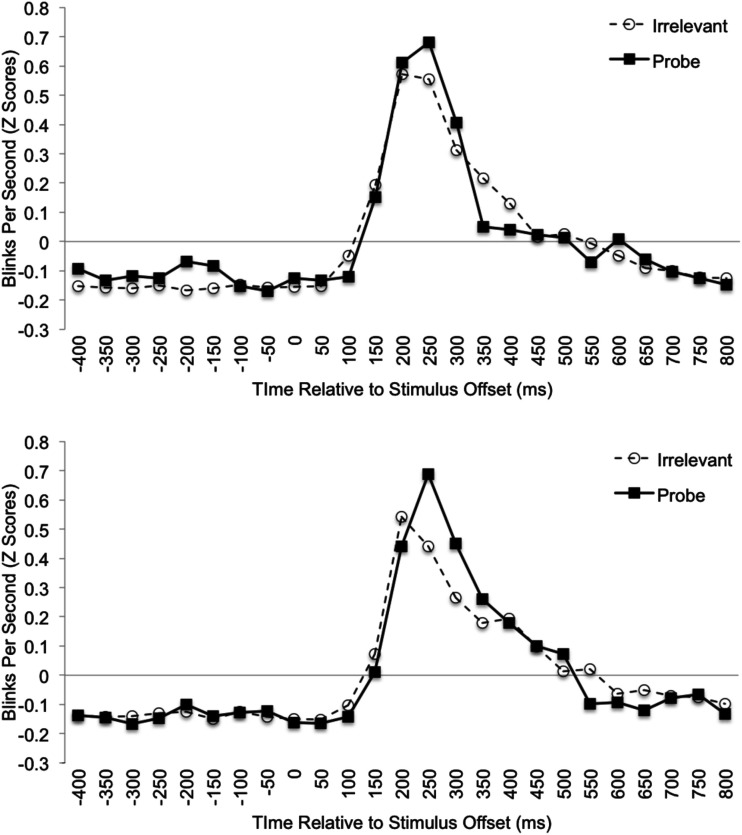
**Mean standardized blink data plotted as a function of Time (50 ms bins relative to stimulus offset) and Stimulus Type for the familiar-probe (top graph) and unfamiliar-probe (bottom graph) conditions**.

**Figure 3:**
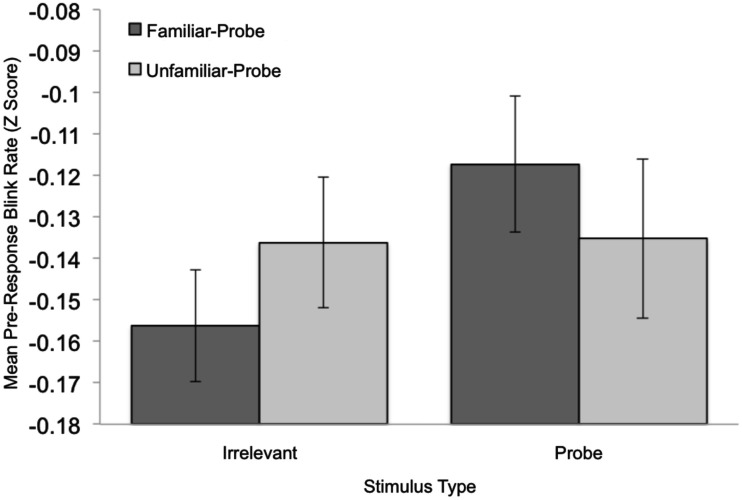
**Mean standardized pre-response (−100 to −400 ms relative to the response) blink-rate data plotted as a function of Stimulus Type and Condition**. Error bars indicate standard error of the mean.

To examine the predicted effects of peak blink-rate, time to reach peak blink-rate, and average blink-rate for the period 200–400 ms post-response, a set of 2 Condition (familiar-probe vs. unfamiliar-probe) × 2 Stimulus Type (probe vs. irrelevant) mixed-model ANOVAs were performed on these measures, but each failed to yield significant main effects or interactions, *F*s < 1. To examine the predicted effect of pre-response blink-rate, we analyzed differences between probe and irrelevant data that can be seen in Figure 2 (top graph) for familiar-probes only, −400 to −100 ms relative to stimulus offset. Mean standardized blink-rates for bins during this period are plotted in Figure 3 as a function of Condition and Stimulus Type. A 2 Condition (familiar-probe vs. unfamiliar-probe) × 2 Stimulus Type (probe vs. irrelevant) mixed-model ANOVA was performed that yielded a main effect of Stimulus Type, *F*(1,50) = 5.60, *p* = 0.02, η^2^ = 0.02, and a Condition × Stimulus Type interaction, *F*(1,50) = 3.78, *p* = 0.06, η^2^ = 0.01, approaching significance. A *post hoc* comparison revealed that in the familiar-probe condition, mean blink-rate during this period was 0.18 (SD = 0.38) b/s higher on probe than irrelevant trials, *t*(29) = 2.63, *p* < 0.05.

#### Pupil analysis

**Figure 4:**
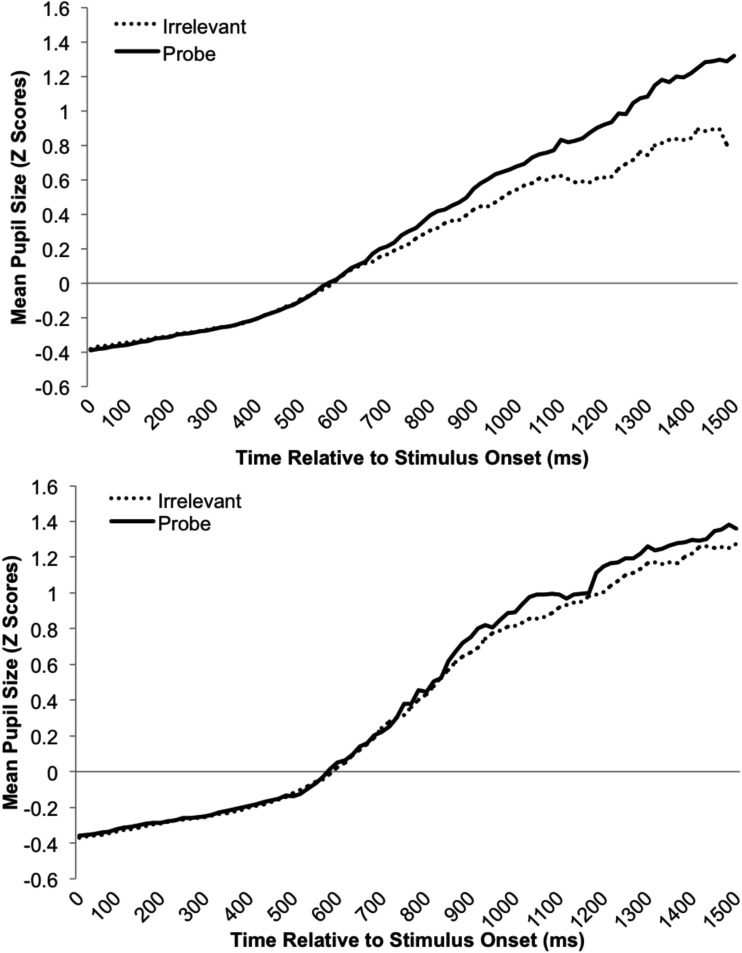
**Mean standardized pupil-size data plotted as a function of time (from stimulus onset to 1500 ms afterward) and stimulus type for the familiar-probe (top graph) and unfamiliar-probe (bottom graph) conditions**.

**Figure 5:**
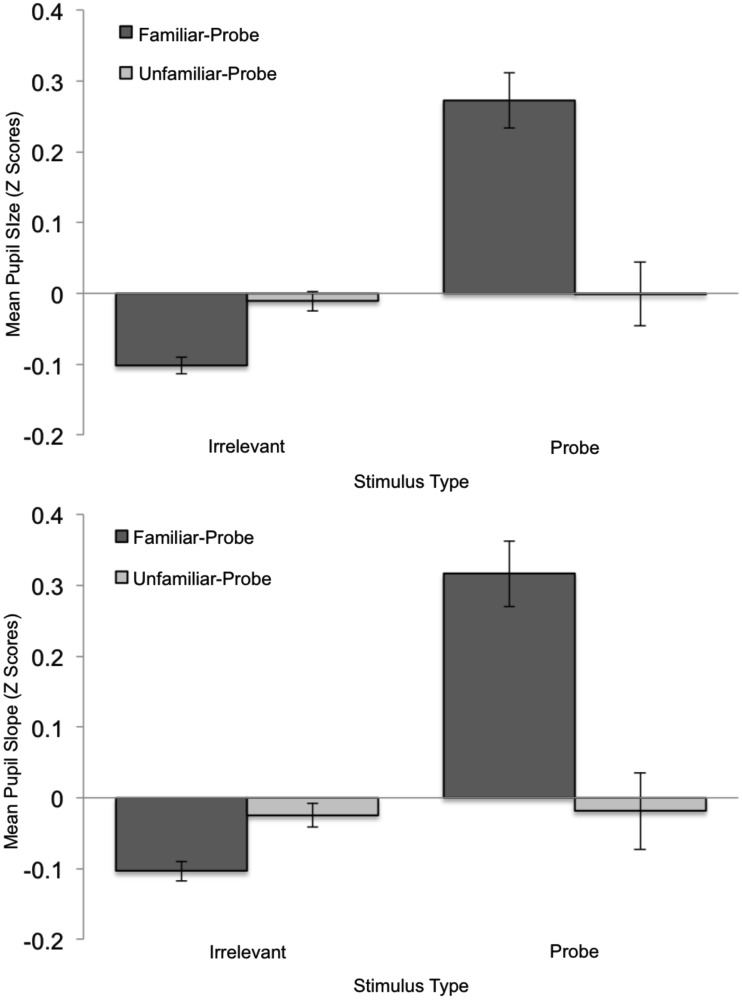
**Mean standardized pupil-size (top graph) and pupil-slope (bottom graph) plotted as a function of Stimulus Type and Condition**. Error bars indicate standard error of the mean.

Figure 4 shows standardized pupil data over time as a function of Stimulus Type and Condition, and allows one to assess the sources of mean pupil and pupil-slope effects. These effects are summarized in Figure 5 which depicts *Z*-Scores for the mean pupil-size data averaged over time as a function of Stimulus Type and Condition (top graph), as well as a similar plot of the pupil-slope data (bottom graph). The goal of the following analysis was to test the prediction that familiar-probe faces would lead to a greater mean pupil-size, and a greater pupil-slope compared to irrelevant faces.

A Condition (familiar-probe vs. unfamiliar-probe) × 2 Stimulus Type (probe vs. irrelevant) mixed-model ANOVA was performed on mean pupil-size with Stimulus Type as the within-subjects variable and revealed a main effect of Stimulus Type, *F*(1,50) = 27.28, *p* < 0.001, η^2^ = 0.01, as well as a Condition × Stimulus Type interaction, *F*(1,50) = 27.93, *p* < 0.001, η^2^ = 0.01. These results verify that mean pupil-size was 0.10 mm (SD = 0.08) greater on probe trials than irrelevant trials, *t*(29) = 6.86, *p* < 0.001, but only when probes were familiar. A similar analysis performed on pupil-slope revealed a main effect of Stimulus Type, *F*(1,50) = 7.73, *p* < 0.01, η^2^ = 0.02, as well as a Condition × Stimulus Type interaction, *F*(1,50) = 23.1, *p* < 0.001, η^2^ = 0.07. This pattern of results is similar to the average pupil result and indicates that pupil-size grew 8% faster when viewing probe than irrelevant faces, *t*(29) = 6.14, p < 0.001, but only in the familiar-probe condition.

#### Classification analysis

The results of the classification analysis for the present data are listed in Table 2 and show that RT led to more accurate classifications than pupil-size, *Z* = 1.77, *p* < 0.05, and slope, *Z* = 2.71, *p* < 0.01. This was not true for RT vs. pre-response blink-rate, *Z* = 1.43, *p* = 0.08. Although combinations of RT and ocular measures produced higher classification rates than tests based on individual ocular measures, all *p* < 0.05, this was likely driven by significant differences between individual RT and pupil measures. Similarly, combining ocular measures did not significantly improve overall classification accuracy compared to pupil-size alone. However the hit rate achieved by combining pupil and blink measures was higher than pupil-size alone, *Z* = 1.81, *p* < 0.05. Bivariate correlations were calculated between RT and various ocular measures; we found that only the RT and pupil-size measures were significantly correlated, *r*(30) = 0.65, *p* < 0.05.

**Table 2:** **Results from the compound classification procedure (variance-ratio and Kolmogorov–Smirnov tests only) for individual and combined measures**.

Predictor	Hit rate	False-alarm rate	Classification accuracy
**INDIVIDUAL MEASURES**			
Manual			
Response time (RT)	0.97	0	98%
Ocular			
Pupil-size	0.83	0	92%
Pupil-slope	0.70	0	85%
Pre-resp. blink-rate	0.90	0.045	93%
**COMBINED MEASURES**			
Manual and ocular			
RT + pupil	1.0	0	100%
RT + slope	0.97	0	98%
RT + blink	1.0	0.045	98%
All (RT + ocular)	1.0	0.045	98%
Ocular			
Pupil + slope	0.90	0	94%
Pupil + blink	0.97	0.045	96%
Pupil + slope + blink	0.97	0.045	96%

## Discussion

The primary goal of the present study was to examine whether RT and eye-based measures could be successfully used to detect concealed knowledge either alone or in combination. Although several studies have previously reported successful RT-based tests, previous ocular-based paradigms have less consistent successes and have yielded a wider range of false-alarm and miss rates. Because multiple aspects of the eyes’ response to a stimulus can be assessed simultaneously using modern eye-trackers, we analyzed pupil-size, pupil-slope, average blink-rate, peak blink-rate, and overall temporal distribution of blinks. To our knowledge, no previous study has simultaneously examined RT and this array of ocular measures in a CKT paradigm.

Participants in this study learned sets of probe and target face pictures and were later asked to respond “yes” to indicate familiarity of target faces and “no” to indicate lack of familiarity with novel irrelevant faces. Participants also responded to probe faces and were asked to respond “no” regardless of whether the probes were the ones previously studied (familiar-probe condition), or whether the probes were novel faces (unfamiliar-probe condition). With this paradigm, we examined the individual and combined diagnosticity of RT, accuracy, and multiple indices of pupil and blink responding. For individual measures we predicted that responsivity would be greater on probe than irrelevant trials, but only in the familiar-probe condition.

### Performance of individual measures

Consistent with predictions, participants were significantly slower and less accurate when responding “no” to familiar-probe faces compared to irrelevants. This pattern of results for RT and accuracy measures is similar to ones previously reported with the CKT paradigm (e.g., Allen et al., 1992; Seymour et al., 2000; Seymour and Kerlin, 2008; Seymour and Fraynt, 2009; Verschuere et al., 2010; Visu-Petra et al., 2011). Based on work by Lubow and Fein (1996), we also predicted that average pupil-size and mean pupil-slope would be greater when responding to probes compared to irrelevants in the familiar-probe condition. Although Lubow and Fein reported a successful test based on mean-pupil size, they only commented on apparent differences in pupil-slope. The present results show that pupil-size grows faster and achieves a greater final size on familiar-probe trial than irrelevant trials. For blinking behavior, numerous predictions were made following Fukuda (2001)’s successful demonstration that the way blinking is distributed over the course of test trials (especially the period before and after the overt response) can discriminate between those with and without concealed knowledge. Unfortunately, an analysis of the overall function relating blinking to time (temporal distribution of blinks) compared across conditions did not reach statistical significance. This was also true for predicted increases in related peak blink-rate and time-to-peak blink-rate measures; these showed no sensitivity to probe-familiarity. Fukuda reported greater increases in blink-rate just prior to the overt response, and also just after the response. In the present data, a similar prediction for the post-response blink-rate was not supported; significant increased blinking was noticed, but this increase was not greater for familiar-probe stimuli. Our final prediction for blinking was based on Fukuda’s pre-response blink-rate finding. Here we did find a small, but statistically significant increase in blinking for familiar-probe trials compared to irrelevants in the period −400 to −100 ms before to the overt response. Interestingly, this increase in blink-rate was most prominently observed in the averaged data for the period between 250 and 100 ms prior to response onset (see Figure 3). The lack of effect during the final 100 ms of this period suggests that pre-response blinks may be indexing a single, late, processing stage associated with concealed knowledge responding and is consistent with a recently proposed response-conflict based model (Seymour and Schumacher, 2009; Schumacher et al., 2010). In their Parallel Task-Set model, Seymour and colleagues offers an account of both the timing of response-conflict in the CKT paradigm and the additional variance in processing observed for familiar-probe trials. Overall, we found that RT, accuracy, pre-response blink-rate, pupil-size, and pupil-slope measures each differentiated responses in the familiar and unfamiliar-probe conditions. To our knowledge, this is the first demonstration of a CKT paradigm simultaneously assessing these measures.

### Compound classification procedure

A CCP comparing probe and irrelevant distributions on shape and variance was used. Significant differences between probe and irrelevant distributions on the basis of shape or variance indicated familiarity with the probe faces. Although this procedure has been used in previous studies (Seymour et al., 2000; Seymour and Kerlin, 2008; Seymour and Fraynt, 2009), the present study is the first to describe this procedure in detail, and the first demonstration of its fitness for data other than RT and Accuracy. The 0.98 classification rate observed with the RT measure was comparable to the 0.92–0.97 rates previously reported using this paradigm (Seymour et al., 2000; Seymour and Kerlin, 2008; Seymour and Fraynt, 2009). Similarly, the pupil-size measure yielded a higher overall classification rate (0.92) here than the 0.66–0.88 rates typically reported (Janisse and Bradley, 1980; Lubow and Fein, 1996). Although tests based on combined measures yielded high classification rates, they were not overall more accurate than using RT in isolation. We note that the failure of compound measures to outperform singular ones was not due to correlations between various measures, as only RT and pupil-size were correlated.

For the pupil-slope measure, it is less clear how to interpret previous studies. Although slope changes were noted previously in Lubow and Fein’s (1996) pupil-size based paradigm, classification accuracy using pupil-slope was not provided. Overall, the performance of the present slope-based analysis was less impressive than those using pupil-size and blink measures. This is more likely to be a result of the relatively low discriminability of the slope measure rather than limitations of the CCP. Although no participants in the unfamiliar-probe condition showed slope differences between probe and irrelevant stimuli, 30% of participants in the familiar-probe condition also failed to show such differences, resulting in a relatively high miss rate. However, the overall 85% classification accuracy provided by the slope measure was equally high as Lubow and Fein’s pupil-size based test.

Although in the present study the overall temporal distribution of blinks did not discriminate familiar-probe and irrelevant trials as in Fukuda’s (2001) study, we did find the predicted difference in mean blink-rate just prior to the response. When analyzed using the CCP, blink-rate yielded an overall classification rate of 0.93, comparable to performance of the pupil-size measure (0.85), and not statistically different than the classification rate using RT (0.98). This was surprising for a mean difference of less than one-quarter blink per second. This result highlights an important advantage of the CCP’s focus on the shape and variance of response distributions instead of a single cutoff value: it is less affected by the distribution overlap if the distributions have different shapes (e.g., Farwell and Donchin, 1991). Leal and Vrij (2010) also examined blink responses during a CKT and found hit and false-alarm rates (0.75 and 0.23, respectively) lower than in the present study (0.90 and 0.045 respectively). One possible source of this difference is the nature of their analysis window; it was only reported that blinks were analyzed “during an arbitrarily defined 10 s window” between stimulus onset and the vocal response. This window would be a super-set of the one analyzed in the present study in which only a small subset proved diagnostic (i.e., the 400 ms just prior to response onset). Thus, it is possible that Leal and Vrij averaged over a relatively small amount of diagnostic and a large amount of non-diagnostic blink data, artificially limiting the accuracy of their classification. If this is the case, then results from the present study and previous studies may yet indicate that blink analysis of concealed knowledge is more promising than previously thought.

### Combined vs. individual measures

Classification analyses were performed to examine the prediction that tests based on multiple measures would outperform those using individual measures. Although most combined measures led to higher detection rates than individual measures, few improvements were statistically significant; one notable exception was found using ocular measures. Although combining measures did not change the false-alarm rate, combining pupil-size and blink-rate measures led to a significantly greater classification than using pupil-size alone. Otherwise, combined tests appeared to offer only minor improvements; most likely due to the strong performance produced by the individual measures (RT in particular). We found a correlation between RT and pupil-size measures, but not between pupil-size and blink measures. This may explain why the RT + pupil combined-measure failed to improve upon RT alone, whereas the pupil + blink measure did. Thus, it appears that the high individual classification accuracy of some individual measures may have constrained the improvement offered by combinations. Similarly, high correlations between ocular and electrodermal measures (e.g., Bradley et al., 2008) suggest that other limitations may exist for combinations involving ocular measures.

### Suggestions for future research

One limitation of the present study is in its ability to consider pupil-size independent of RT. This is due to the fact that trials ended immediately following the overt manual response. Because collection of pupil data also ended on each trial concomitant with the response, it is possible that the pupil-size based concealed knowledge effect is solely an indication of the larger RTs on familiar-probe trials relative to irrelevant trials. The significant correlation between RT and pupil-size supports this alternative explanation. Follow-up studies that lack an overt response, or in which the collection of pupil data continues for some time following the response, would be informative. However, Lubow and Fein’s (1996) report of successful pupil-size based CKTs without response-terminated pupil recording tempers this interpretation somewhat. Furthermore, the presence of pupil-slope effects here (which were not correlated with RT) and in Lubow and Fein’s study suggests that average pupil-size differences between probe and irrelevant trials are not merely a result of the passage of time. Despite these caveats, further investigation is warranted. One interesting alternative for avoiding dependence on overt responses is to use more complex stimuli (e.g., sentences or picture arrays) and examine ocular scan-patterns during the CKT (e.g., Webb et al., 2009a,b; Kircher et al., 2010; Cook et al., 2012).

Another issue for further study involves a detailed comparison of the CCP with the diverse range of previously reported CKT classification procedures. For example, the implications of using correlated measures of underlying response distribution morphology, the effect of the corrected alphas, and the influence of the Fishers randomization procedure would need to be modeled statistically to properly distinguish the CCP from related techniques such as the Independent Parallel Testing procedure (IPT; National Research Council, 2003), discriminant-function analysis (e.g., Nose et al., 2009), and logistic regression analysis (e.g., Gamer et al., 2006; Gamer, 2011). Of particular interest is how exactly hit rates and false-alarms are affected by each additional CCP sub-test. It is also unknown whether the CCP offers a significant advance over straightforward modifications to established approaches such as ROC analysis (Green and Swets, 1966; Bamber, 1975; Hanley, 1982). Such comparisons with the CCP would need to consider its primary design feature; reliance on generic differences between response distributions. This focus on only ways in which two distributions may vary in CKT-related paradigms (deviation, skewness, and in some cases number of observations; c.f., Seymour and Schumacher, 2009) means that there is no need to vary classification parameters between tests, even if test parameters or subject demographics change. Unlike some discriminant-function based procedures, its fitness is not based on a limited set of previously observed data. Thus, the only parameter that *can* change is the nominal alpha for the constituent statistical tests, and this would only be justifiable if the test were made more strict, but not less. Such a change would be in service of an even lower tolerance of false-alarms than offered by the standard alpha level of 0.05, and not the nature of the underlying test.

The closest alternative to the CCP is the IPT approach, however, the constituent tests can be anything, and the cutoffs used for these tests may vary from one use to the next. For example, Meijer et al. (2007) reported such a procedure for successfully combining performance on a skin-conductance based CKT with performance on a test of malingering. Although each test used standard task-specific cutoffs to classify subjects prior to the combined classification using IPT, such classifications could have been decided using a number of potential decision policies; each having a potential impact on this test’s sensitivity and specificity (National Research Council, 2003). In contrast, the constituent tests for the CCP are always statistical hypothesis tests; combining measures occurs prior to classification and results in two distributions of combined scores (one for probes and one for irrelevants) that are then compared statistically. Thus, it may be useful to investigate whether or not an IPT modified to accept the raw score from individual or combined CKT measures would be effectively similar to the CCP.

The present work has implications for applied work in forensic settings. However, an important next step in this research is to examine combined efficiency of ocular and RT measures in paradigms using mock-crimes that facilitate variable probe encoding, and longer retention intervals that would allow for forgetting or interference (e.g., Carmel et al., 2003). Such manipulations have previously been shown to modulate the effectiveness of the RT measure and may provide more room for the contribution of simultaneous ocular measures along with RT (Seymour and Fraynt, 2009). Such research may also employ explicit countermeasure instructions to manipulate the motivation to “beat the test.” Countermeasure manipulations are sometimes sufficient to attenuate the RT-based concealed knowledge effect (e.g., Rosenfeld et al., 2004), but not always (Seymour et al., 2000; Seymour and Fraynt, 2009). Even with such manipulations, it may be possible that conducting CKT research using undergraduate populations who lack the intrinsic motivation to deceive found in applied contexts limits the generalizability of our results. However, despite larger effect-sizes on average for laboratory settings compared to tests in the field, such differences do not always affect classification accuracy. For example, a study by Pollina et al. (2004) showed that classification accuracy was similar in laboratory and field-tests, despite differences in effect-sizes. Similarly, in a meta-analysis of CKT studies reported by Ben-Shakhar and Elaad (2003), it was shown that significant differences in test effect-sizes resulted when highly motivated participants (*d* = 1.76) were compared to those with low motivation (*d* = 1.34). However, this disparity failed to result in differences in respective test efficiencies (*a* = 0.82 and 0.80 respectively, for high and low motivation participants).

## Conflict of Interest Statement

The authors declare that the research was conducted in the absence of any commercial or financial relationships that could be construed as a potential conflict of interest.
